# Association of Public Interest in Preventive Measures and Increased COVID-19 Cases After the Expiration of Stay-at-Home Orders: A Cross-Sectional Study

**DOI:** 10.1017/dmp.2020.333

**Published:** 2020-09-10

**Authors:** Micah Hartwell, Benjamin Greiner, Zach Kilburn, Ryan Ottwell

**Affiliations:** Oklahoma State University Center for Health Sciences, Department of Psychiatry and Behavioral Sciences, Tulsa, OK; University of Texas Medical Branch, Department of Internal Medicine, Galveston, TX; Kilburn Consulting, LLC, Tulsa, OK; Oklahoma State University Center for Health Sciences, College of Osteopathic Medicine, Tulsa, OK

**Keywords:** COVID-19, public awareness, public preparedness, search trends

## Abstract

**Objective:**

Following stay-at-home (SAH) orders issued for coronavirus disease (COVID-19), state-level economic concerns increased and many let these orders expire. As a method to measure public preparedness, we sought to explore the association between public interest in preventive measures and the easing of SAH orders – specifically the increases in COVID-19 cases and fatalities after the orders expired.

**Methods:**

Search volume was collected from Google Trends for “hand sanitizer,” “social distancing,” “COVID testing,” and “contact tracing” for each state. Bivariate correlations were computed to analyze associations between public interest in preventive measures, changes in confirmed COVID-19 cases after SAH expirations, COVID-19 case-fatality rates, and by-state presidential voting percentages.

**Results:**

A higher interest in preventive measures was associated with lower rates of confirmed cases after SAH orders had expired (r = −0.33), higher state-wide deaths per capita (r = 0.42), and case-fatality rates (r = 0.60). Moderate to strong negative correlations were found between states’ percentage of voters supporting the Republican nominee in 2016 and proportion of queries for average preventive measures (r = −0.77).

**Conclusion:**

Our investigation shows that increased public interest in COVID-19 prevention was associated with longer SAH orders and less COVID-19 cases after the SAH orders’ expiration; however, it was also associated with higher case-fatality rates.

The influence of the 2019 coronavirus disease (COVID-19) on the world has been substantial and continues to affect US citizens’ physical and financial health negatively.^1,2^ Although the case-fatality rate for COVID-19 is much lower than other coronaviruses, COVID-19 has killed more people than severe acute respiratory syndrome (SARS) and Middle East respiratory syndrome combined.^3^ Furthermore, infection rates have been climbing across some US states as stay-at-home (SAH) orders have been lifted.^4^ Therefore, it is evident that efforts must be made to halt the resurgence of severe acute respiratory syndrome coronavirus 2 (SARS-CoV-2), the virus responsible for COVID-19, and to re-evaluate the current pandemic preparedness response plans.

As a result of recurring pandemics in the 1900s and early 2000s, such as the 2002–2004 SARS and H5N1 influenza pandemics, efforts were made by the World Health Organization (WHO) to improve understanding of outbreak frameworks and virus transmissibility, thereby resulting in the 2005 WHO global pandemic plan.^5^ Yet, the WHO efforts focused primarily on surveillance. Thus, the Department of Health and Human Services developed the 2006 Pandemic and All-Hazards Preparedness Act to understand pandemic response plans beyond surveillance.^6^ All combined efforts ultimately led to the Centers for Disease Control and Prevention (CDC) 2014 preparedness and response framework for pandemics, which detailed the 8 domains of response planning within an outbreak: incident management, surveillance and epidemiology, laboratory, community mitigation, medical care and countermeasures, vaccine, risk communications, and state/local coordination.^5^

Although pandemic response planning and preparedness have been a rapidly evolving field, room for improvement remains and is highlighted by the current pandemic and the resurgence of patients with COVID-19 in some states. One method of improving outbreak preparedness is through increasing public interest and awareness in preventive measures. For example, a survey of Sierra Leone citizens was conducted during the 2014–2015 Ebola outbreak and found that awareness of the disease was high, yet only 40% knew that isolating themselves from Ebola patients could prevent transmission, an effect that likely worsened the outbreak.^7^

Self-isolation has been necessary during the COVID-19 pandemic and has helped slow the virus transmission. A previous study identified an inverse association between by-state public interest in COVID-19 preventive measures and the timely issuance of SAH orders, which suggests that increasing public interest in preventive measures may improve the timeliness of necessary quarantine orders.^8^ To expand on this previous study, we sought to explore the association between public interest in preventive measures, easing of SAH orders, and increases in COVID-19 cases after their expiration. Finally, we aimed to determine whether public interest in preventive measures was associated with partisan interests.

## METHODS

Methodology from this study was originally developed by Greiner et al.^8^ and modified for this research. Public interest in COVID-19 preventive measures was defined as a relative Internet search volume for prespecified queries, which has been used as a proxy in previous public health investigations.^7^

The state level search volume was collected from Google Trends (https://trends.google.com/trends/), the most widely used search engine for health information by the general public.^9^ Google Trends uses a search volume scale of 0 to 100, with 100 representing the greatest proportion of queries (POQ) in the defined time period. Search topics were selected from the US Surgeon General recommended practices and included “hand sanitizer,” “social distancing,” and “COVID testing.” We also collected a relative search interest for “contact tracing” due to its novelty to the public and increased media attention, in tracking this as a scale pandemic. Subsequently, a composite variable, *average of preventive measures*, was calculated as the mean POQ for “hand sanitizer,” “social distancing,” “COVID testing,” and “contact tracing,” for each state. These queries were analyzed from May 1, 2020, to May 31, 2020, a time frame in which a majority of states’ SAH orders were expiring. Next, we identified the expiration date of each state’s SAH order from the National Academy of State Health Policy (www.nashp.org/governors-prioritize-health-for-all) and compiled the number of positive COVID-19 cases during the 1 week prior to and the third week following the end of the SAH order from the CDC COVID-19 Data Tracker (www.cdc.gov/covid-data-tracker/). In cases where the state did not issue a firm SAH order, the first recognized day of reopening of non-essential businesses will be used. This time delay allows for the estimated 14-day window of exposure to the detection of confirmed cases. We then calculated the percent change in cases within each state. We also calculated the number of days after April 15, 2020, that each state’s SAH order was in effect; the earliest SAH order’s expiration was April 21, 2020. We also extracted total positive COVID-19 cases and deaths per state and calculated the case-fatality rate. Finally, to assess partisan interest, we included the percentage of each state’s constituents voting for the 2016 Republican presidential nominee. Republican voters were selected as opposed to Democratic voters due to recent evidence indicating the former search for COVID-19 information less frequently and have lower perceived risk of the disease.^10^

### Statistical Analysis

We constructed bivariate correlations to analyze associations between public interest in the average of preventive measures, changes in confirmed COVID-19 cases after SAH expirations, COVID-19 cases and deaths per capita, COVID-19 case-fatality rates, population density, and voting percentages by state. Data were compiled on June 30, 2020, and the statistical analysis was completed on July 1, 2020, using Stata 16.1 (StataCorp, College Station, TX). This study does not meet the criteria for human subjects research and was not subject to an Institutional Review Board approval.

## RESULTS

**FIGURE 1:**
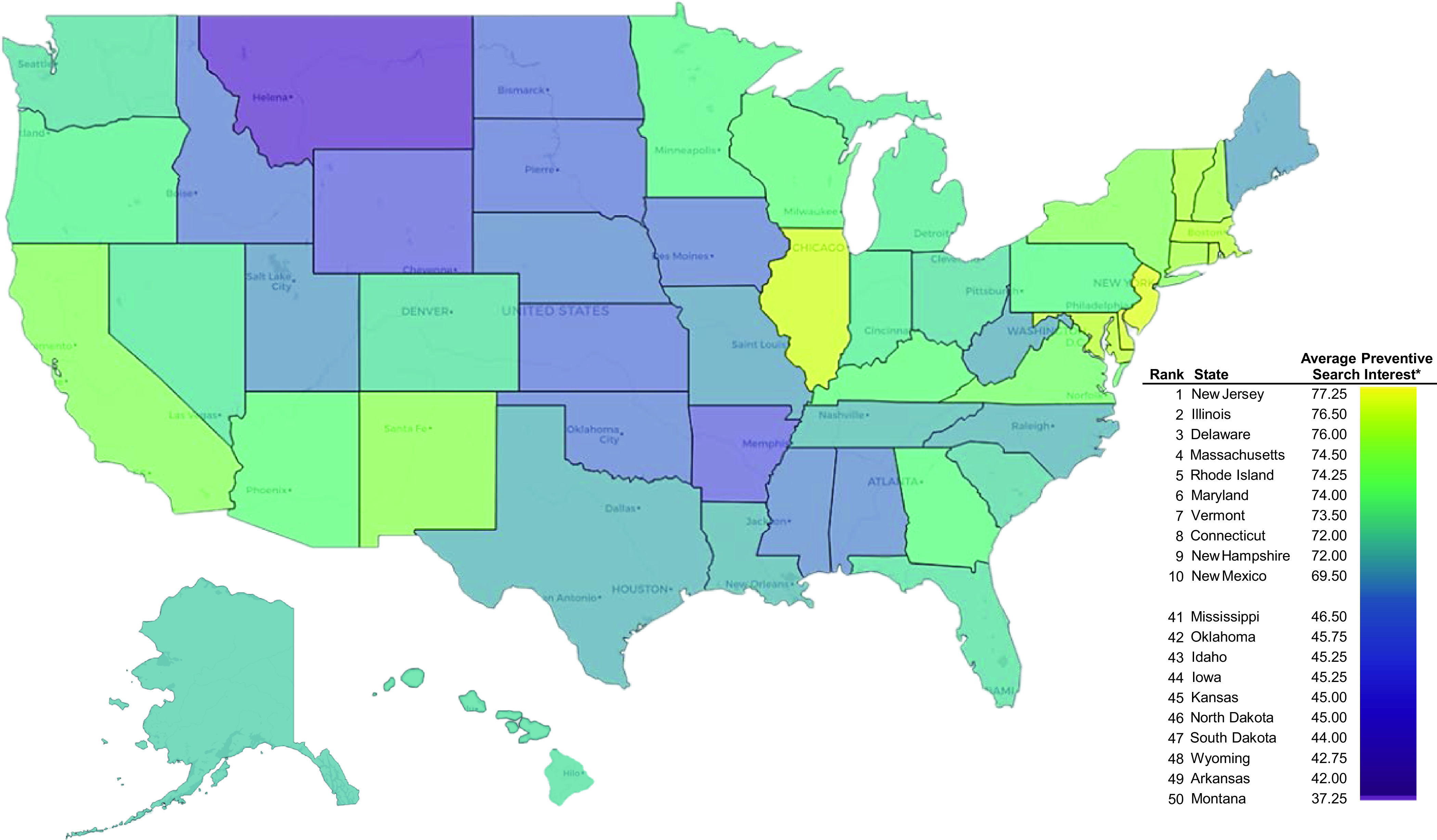
By-state Search Interest in COVID-19 preventive measures.

**FIGURE 2:**
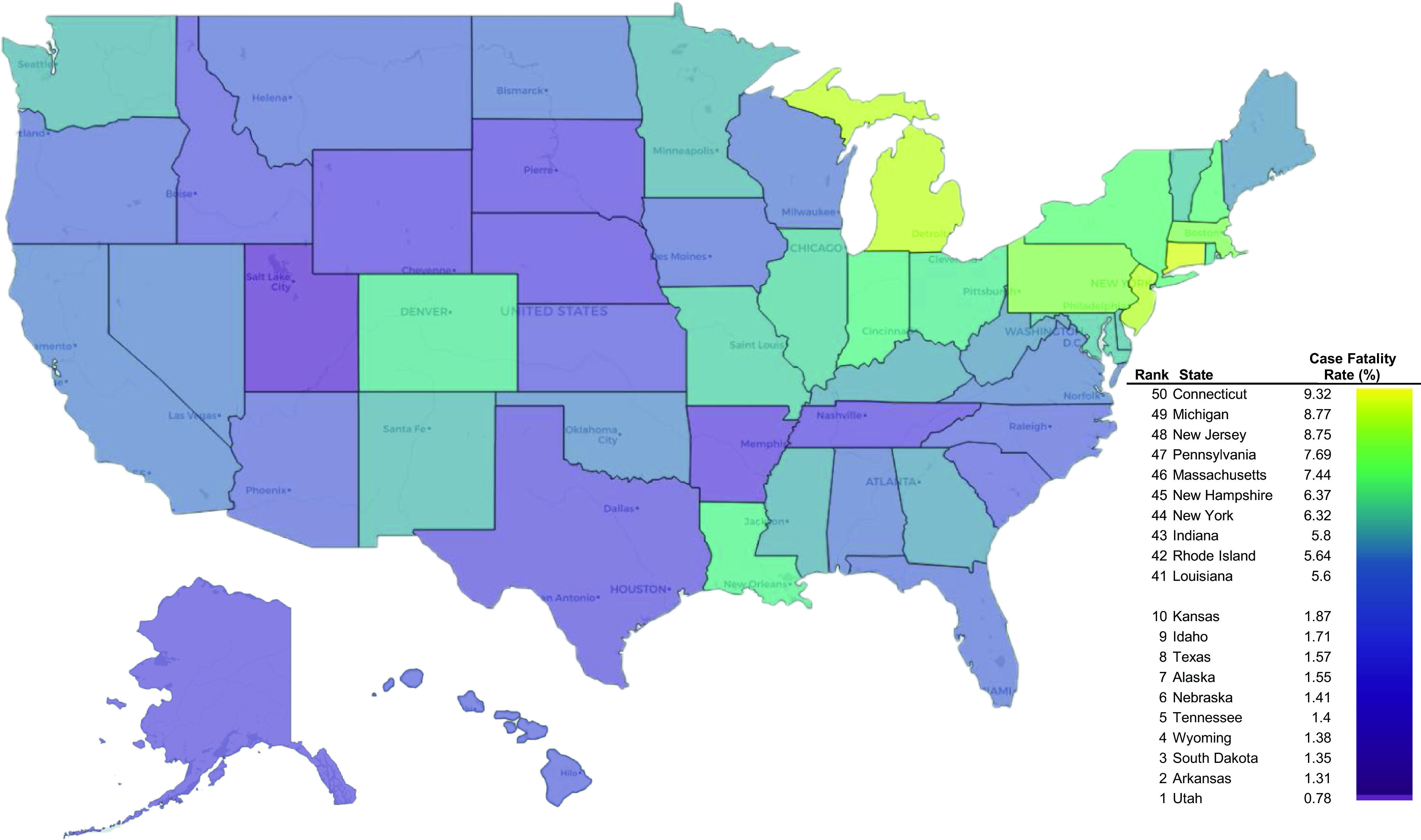
Case-fatality rates of COVID-19 by state with highest and lowest rankings.

Results from our study showed that states in the Northeast and Illinois yielded the highest POQ in the average preventive measures for COVID-19 (Figure 1). A higher interest in preventive measures was associated with lower rates of confirmed cases after official SAH orders had expired (r = −0.33), higher state-wide COVID-19 deaths per capita (r = 0.42), case-fatality rates (r = 0.60), as well as a longer duration of SAH orders past April 15 (r = 0.60; Table 1). Rankings of case-fatality rates by state are shown in Figure 2. The change in positive cases of COVID-19 from the week prior to the expiration of SAH orders to 3 weeks thereafter was the lowest in Massachusetts and Connecticut; this decreased these states’ weekly rates by 290% and 255%, respectively, whereas Arkansas, Hawaii, and Vermont had the highest increases at 72%, 87%, and 93%, respectively (Supplementary Table). While population density was not associated with a state’s total case count at the end of June 2020 (r < 0.01), it was associated with a higher public interest in preventive measures (r = 0.64) and with lower percent changes in case counts after the expiration of SAH orders (r = −0.49). Moderate to strong negative correlations were found between the states’ percentage of voters supporting the Republican nominee in 2016 and POQ for average preventive measures (r = −0.77), COVID-19 deaths per capita (r = −0.37) and case-fatality rates (r = −0.37), and the extended duration of SAH order expirations (−0.52).

**TABLE 1:** Correlations of Preventive Measures Associated With COVID-19, Contact Tracing, and Increased Cases and Deaths Per Capita

	Average of Preventive Measures (APM)^a^	Delay in SAH Expira-tion^b^	Change in Cases After SAH^c^	Total Cases Per Capita^d^	Total Deaths Per Capita^d^	Case-Fatality Rate^d^	Republican Voting^e^	Popul. Density
Delay in SAH expiration^b^	0.55							
Change in cases after SAH^c^	−0.32	−0.07						
Total cases per capita^d^	−0.06	−0.10	−0.02					
Total deaths per capita^d^	0.42	0.21	−0.49	0.62				
Total case-fatality rate^d^	0.60	0.56	−0.42	−0.14	0.54			
Republican voting^e^	−0.74	−0.51	0.13	−0.09	−0.37	−0.37		
Population density	0.64	0.29	−0.49	0.00	0.61	0.58	−0.48	
Previous APM^f^	0.54	0.32	−0.24	0.00	0.18	0.30	−0.73	0.25

a Updated average of preventive measures from search interest in hand sanitizing, COVID-19 testing, social distancing, and contact tracing.

b Cases and deaths as of June 30, 2019.

c Stay-at-home orders, duration in days extending from April 15, 2020.

d By-state change in positive cases from the 7 days prior to the expiration of SAH orders and the third week after expiration.

e Republican voting (%) from the 2016 presidential election results.

f Previously published APM from prior to issuance of stay-at-home orders.

## DISCUSSION

Our study demonstrates that increased public interest in preventive measures is correlated with later SAH expiration dates as well as decreased rates of new COVID-19 cases after the expiration of SAH orders. Furthermore, states with greater interest in prevention were also more likely to have incurred higher COVID-19 deaths per capita and higher case-fatality rates regardless of overall cases per capita. This association between deaths and POQ of preventive measures likely coincides with actions taken in these areas to prevent further spread to prevent hospitals from being over their capacity. Further, states with the highest percentage of Republican voting were less likely to search for COVID-19 preventive measures and also have a shorter duration of SAH orders past April 15; however, interestingly, they were also less severely affected by COVID-related deaths and have lower case-fatality rates. This association between the states’ partisan leaning and COVID-19 severity may help explain the perceived political divide in the necessity of preventive measures, such as wearing face coverings. To our knowledge, our study is the first of its kind to investigate the relationship between the public interest in preventive measures and increasing cases of COVID-19 after the cessation of SAH orders. Here, we discuss key implications of our results, provide recommendations with rationale aimed to increase public interest in COVID-19 safety, and, ultimately, decrease the incidence of new cases.

Public interest in a nationwide pandemic response is necessary for the appropriate dispersal of public health communications, and the lack thereof in the United States is likely impacting the SARS-CoV-2 transmission rate. For example, the United States took 52 days from the first confirmed case to substantially increase COVID-19 public interest compared with 15 days in countries with more successful outbreak forecasts.^11^ Although self-reported preventive measures, such as mask wearing, continue to be as high as 74% within the United States,^12^ poor interest in preventive measures in certain states may result in decreasing adherence to public health recommendations. Thus, our study highlights the need for a unified national response and public policy measures to increase public interest in COVID-19 preventive measures.

Solutions to improve public interest should be multifactorial and represent multiple socio-ecological levels to improve the successfulness of implementation. One method to incorporate intrapersonal, interpersonal, and community ecological levels is to exploit the widespread use of social media platforms. For example, new research has shown that the use of digital interventions, such as texting apps, games, e-mails, and social media, can significantly impact health behavior change.^13,14^ Second, policy-makers must incorporate and use public health experts in their advisory board prior to constructing COVID-19 legislature. Additionally, policy-makers should use grassroots efforts to improve public interest in preventive measures to appropriately allocate community resources.

This study had several strengths and weaknesses. This study’s strengths lie in our robust and previously validated methodology and the use of the most widely used health information search engine. Alternatively, true knowledge and understanding of preventive measures could not be ascertained, and lasting knowledge of preventive measures was likely lower than relative search interests – a potential weakness. Further, this study was cross-sectional and, thus, cause-effect relationships could not be derived. Prospective and interventional studies should be performed in the future to better understand the complexities of how public interest in preventive measures influences nationwide pandemic responses. Additionally, multiple confounding variables, including societal and economic factors, may have impacted the association of increased public interest in preventive measures and higher case-fatality rates. For instance, due to the population density of New York, it may have been more widely understood that preventive measures against infectious diseases were necessary and, thus, persons dwelling within that state may have had increased baseline interest in researching preventive measures while still having higher infection rates. Further, in larger cities with greater spread of the virus, higher case-fatality rates may be associated with hospitals being at or over capacity, while other states were able to adequately secure additional hospital beds and supplies. Alternatively, the economic stability of individual states may have necessitated an earlier reopening to support social services that may have impacted infection and case-fatality rates.

## CONCLUSIONS

Our investigation shows that increased public interest in COVID-19 prevention was associated with longer SAH orders and less COVID-19 cases after the SAH orders’ expiration; however, it was also associated with higher case-fatality rates. Conversely, Republican leaning states had lower public interest in COVID-19 prevention and shorter SAH orders, while also having less severe COVID-19 outcomes.

## Supplementary material

For supplementary material accompanying this paper visit https://doi.org/10.1017/dmp.2020.333. click here to view supplementary material
