# Supplementary material for: Association of Public Interest in Preventive Measures and Increased COVID-19 Cases After the Expiration of Stay-at-Home Orders: A Cross-Sectional Study
Source: Disaster Med Public Health Prep. 2020 Sep 10:1–5. doi: 10.1017/dmp.2020.333 (PMC7642507; doi:10.1017/dmp.2020.333)
Supplement: Supplementary file 1 [file S193578932000333Xsup001.docx]

| Supplementary Table 1. By-state POQ in average preventive measure for COVID-19, COVID-19 cases and deaths, and changes in cases after the expiration of Stay-at-Home orders and rankings. | | | | | | | | | | | |
| --- | --- | --- | --- | --- | --- | --- | --- | --- | --- | --- | --- |
| State | Average Preventive Measures | Rank in Ave. Preventative Measures | Cases per Capita* | Deaths per Capita* | Case Fatality Rate | Rank in Case Fatality Rate | SAH Expiration** | Cases Week Before SAH Expiration | Cases 3rd Week After SAH Expiration | Change in Cases (%) | Rank in Change in Cases |
| Alabama | 47 | 12 | 7.58 | 18.95 | 2.5 | 20 | 4/30/2020 | 1187 | 2724 | 56.42% | 43 |
| Alaska | 55 | 21 | 1.24 | 1.91 | 1.55 | 7 | 4/21/2020 | 36 | 16 | -125.00% | 8 |
| Arizona | 63.5 | 36 | 10.24 | 21.82 | 2.13 | 14 | 5/15/2020 | 2209 | 2146 | -2.94% | 23 |
| Arkansas | 42 | 2 | 6.71 | 8.78 | 1.31 | 2 | 5/4/2020 | 347 | 1224 | 71.65% | 45 |
| California | 69.25 | 39 | 5.48 | 15.02 | 2.74 | 22 | 5/12/2020 | 10567 | 16009 | 33.99% | 38 |
| Colorado | 57.75 | 26 | 5.61 | 29.1 | 5.19 | 39 | 4/26/2020 | 3238 | 1854 | -74.65% | 13 |
| Connecticut | 72 | 42 | 13 | 121.17 | 9.32 | 50 | 5/20/2020 | 3553 | 1002 | -254.59% | 2 |
| Delaware | 76 | 48 | 11.68 | 52.07 | 4.46 | 34 | 5/31/2020 | 533 | 196 | -171.94% | 4 |
| Florida | 57.25 | 25 | 6.81 | 16.51 | 2.42 | 17 | 4/30/2020 | 3516 | 3959 | 11.19% | 31 |
| Georgia | 63 | 34 | 7.48 | 26.22 | 3.51 | 28 | 4/30/2020 | 4008 | 4016 | 0.20% | 25 |
| Hawaii | 57.75 | 27 | 0.63 | 1.27 | 2 | 11 | 5/31/2020 | 8 | 63 | 87.30% | 46 |
| Idaho | 45.25 | 7 | 2.98 | 5.09 | 1.71 | 9 | 4/30/2020 | 148 | 193 | 23.32% | 36 |
| Illinois | 76.5 | 49 | 11.33 | 56.05 | 4.95 | 38 | 5/31/2020 | 8243 | 4368 | -88.71% | 11 |
| Indiana | 59.75 | 29 | 6.72 | 38.98 | 5.8 | 43 | 5/1/2020 | 4235 | 2659 | -59.27% | 14 |
| Iowa | 45.25 | 8 | 9.12 | 22.44 | 2.46 | 18 | 4/30/2020 | 2700 | 2190 | -23.29% | 21 |
| Kansas | 45 | 5 | 4.96 | 9.27 | 1.87 | 10 | 5/3/2020 | 1702 | 761 | -123.65% | 9 |
| Kentucky | 63.25 | 35 | 3.44 | 12.53 | 3.65 | 29 | 5/20/2020 | 989 | 946 | -4.55% | 22 |
| Louisiana | 53.25 | 20 | 12.28 | 68.81 | 5.6 | 41 | 5/15/2020 | 2420 | 2483 | 2.54% | 27 |
| Maine | 49.75 | 14 | 2.39 | 7.81 | 3.26 | 27 | 5/31/2020 | 251 | 197 | -27.41% | 18 |
| Maryland | 74 | 45 | 11.12 | 52.52 | 4.72 | 36 | 5/15/2020 | 5452 | 3427 | -59.09% | 15 |
| Massachusetts | 74.5 | 47 | 15.65 | 116.48 | 7.44 | 46 | 5/18/2020 | 7720 | 1977 | -290.49% | 1 |
| Michigan | 59.75 | 30 | 7.03 | 61.69 | 8.77 | 49 | 5/28/2020 | 1722 | 1757 | 1.99% | 26 |
| Minnesota | 61 | 32 | 6.36 | 26.07 | 4.1 | 31 | 5/17/2020 | 3637 | 1477 | -146.24% | 7 |
| Mississippi | 46.5 | 10 | 8.93 | 35.58 | 3.99 | 30 | 5/11/2020 | 1467 | 1518 | 3.36% | 28 |
| Missouri | 49.75 | 15 | 3.43 | 16.26 | 4.74 | 37 | 5/3/2020 | 1215 | 974 | -24.74% | 20 |
| Montana | 37.25 | 1 | 0.86 | 2.06 | 2.39 | 15 | 4/24/2020 | 18 | 13 | -38.46% | 17 |
| Nebraska | 46.75 | 11 | 9.77 | 13.8 | 1.41 | 6 | 5/4/2020 | 2552 | 1771 | -44.10% | 16 |
| Nevada | 58.25 | 28 | 5.81 | 16.36 | 2.82 | 24 | 5/9/2020 | 602 | 771 | 21.92% | 35 |
| New Hampshire | 72 | 43 | 4.24 | 26.99 | 6.37 | 45 | 5/31/2020 | 396 | 153 | -158.82% | 6 |
| New Jersey | 77.25 | 50 | 19.28 | 168.79 | 8.75 | 48 | 5/18/2020 | 7296 |  |  |  |
| New Mexico | 69.5 | 41 | 5.63 | 23.46 | 4.17 | 33 | 5/15/2020 | 830 | 897 | 7.47% | 30 |
| New York | 69.25 | 40 | 20.2 | 127.7 | 6.32 | 44 | 5/15/2020 | 12691 | 4684 | -170.94% | 5 |
| North Carolina | 52.25 | 18 | 6.05 | 12.63 | 2.09 | 13 | 5/8/2020 | 2359 | 5478 | 56.94% | 44 |
| North Dakota | 45 | 6 | 4.64 | 11.55 | 2.49 | 19 | 5/1/2020 | 304 | 164 | -85.37% | 12 |
| Ohio | 57 | 23 | 4.37 | 24.11 | 5.52 | 40 | 5/29/2020 | 3158 | 3920 | 19.44% | 34 |
| Oklahoma | 45.75 | 9 | 3.33 | 9.73 | 2.92 | 25 | 5/6/2020 | 583 | 463 | -25.92% | 19 |
| Oregon | 60 | 31 | 2.01 | 4.84 | 2.4 | 16 | 5/12/2020 | 370 | 587 | 36.97% | 39 |
| Pennsylvania | 61.75 | 33 | 6.72 | 51.66 | 7.69 | 47 | 6/4/2020 | 3221 | 4360 | 26.12% | 37 |
| Rhode Island | 74.25 | 46 | 15.82 | 89.3 | 5.64 | 42 | 5/8/2020 | 1490 | 690 | -115.94% | 10 |
| South Carolina | 56.25 | 22 | 6.73 | 13.98 | 2.08 | 12 | 5/4/2020 | 1013 | 1683 | 39.81% | 40 |
| South Dakota | 44 | 4 | 7.59 | 10.29 | 1.35 | 3 |  |  |  |  |  |
| Tennessee | 52.5 | 19 | 6.19 | 8.66 | 1.4 | 5 | 4/30/2020 | 2009 | 2345 | 14.33% | 33 |
| Texas | 51.75 | 17 | 5.28 | 8.29 | 1.57 | 8 | 4/30/2020 | 5281 | 5653 | 6.58% | 29 |
| Utah | 48.75 | 13 | 206.49 | 160.13 | 0.78 | 1 | 5/1/2020 | 880 | 864 | -1.85% | 24 |
| Vermont | 73.5 | 44 | 0.38 | 1.75 | 4.64 | 35 | 5/15/2020 | 6 | 83 | 92.77% | 47 |
| Virginia | 66.5 | 38 | 7.29 | 20.39 | 2.8 | 23 | 6/10/2020 | 4321 |  |  |  |
| Washington | 57 | 24 | 4.17 | 17.2 | 4.13 | 32 | 5/31/2020 | 1547 | 2630 | 41.18% | 42 |
| West Virginia | 51.25 | 16 | 1.6 | 5.19 | 3.24 | 26 | 5/3/2020 | 128 | 215 | 40.47% | 41 |
| Wisconsin | 64 | 37 | 5.33 | 13.47 | 2.53 | 21 | 5/13/2020 | 1828 | 2073 | 11.82% | 32 |
| Wyoming | 42.75 | 3 | 2.51 | 3.46 | 1.38 | 4 | 5/1/2020 | 210 | 71 | -195.77% | 3 |
| *As reported through 6/30/2020 | | | | | | | | | | | |
| **Stay at home order expiration extracted from https://www.nashp.org/governors-prioritize-health-for-all/. | | | | | | | | | | | |
